# CT findings of basaloid squamous cell carcinoma of the lung in 12 patients: A distinct category of squamous cell carcinoma in 2015 WHO classification of lung tumors

**DOI:** 10.1097/MD.0000000000029197

**Published:** 2022-05-20

**Authors:** Chu Hyun Kim, Yoon Ki Cha, Joungho Han, Jun Ho Kim, Tae Jung Kim, Myung Jin Chung, Jung Hee Lee, Hyun Jung Yoon

**Affiliations:** a Center for Health Promotion, Samsung Medical Center, Seoul, Republic of Korea; b Department of Radiology and Center for Imaging Science, Samsung Medical Center, Sungkyunkwan University School of Medicine, Seoul, Republic of Korea; c Department of Pathology, Samsung Medical Center, Sungkyunkwan University School of Medicine, Seoul, Republic of Korea; d Department of Thoracic & Cardiovascular Surgery, Samsung Medical Center, Sungkyunkwan University, School of Medicine, Seoul, Republic of Korea; e Department of Radiology, Veterans Health Service Medical Center, Seoul, Republic of Korea.

**Keywords:** computed tomography, lung cancer, squamous cell carcinoma, X-ray

## Abstract

Basaloid squamous cell carcinoma (SCC) is very rare subtype of SCC of the lung and it is important to distinguish basaloid to other subtypes of SCCs, since the prognosis of basaloid subtype is considered poorer than that of other non-basaloid subtypes of SCCs. Aim of this study was to assess computed tomography (CT) findings of basaloid SCC of the lung in 12 patients.

From January 2016 to April 2021, 12 patients with surgically proven basaloid SCC of the lung were identified. CT findings were analyzed, and the imaging features were compared with histopathologic reports. Clinical and demographic features were also analyzed.

Axial location of the tumor was central in 5 patients, while 7 was in peripheral. Of the 7 patients whose tumors were located in the peripheral, margin of the tumor were smooth (*n* *=* 2), lobulated (*n* *=* 2), or spiculated (*n* *=* 3). After contrast injection, net enhancement value ranged from 15.8 to 71.8 HU (median, 36.4 HU). Endobronchial growth were seen in 5 patients and these patients accompanied obstructive pneumonia or atelectasis. Internal profuse necrosis, cavitation, or calcifications were not seen.

On CT, basaloid squamous cell presents as solitary nodule or mass with moderate enhancement. Tumor was located either peripheral or central compartment of the lung and cavitation was absent.

## 1 Introduction

In the latest 2015 World Health Organization (WHO) classification of tumors of the lungs,^[1]^ basaloid carcinoma is now categorized as a subtype of squamous cell carcinoma (SCC). Prior to the 2015 WHO classification, basaloid carcinomas were considered as variant of large cell or SCCs, since pathologic features simulate these carcinomas.^[2,3]^ However, after basaloid carcinomas were recognized to express squamous markers, they were excluded from the subtype of large cell carcinoma and were re-categorized as a subtype of SCC. Therefore, subtypes of SCC now consists of keratinizing, non-keratinizing, and basaloid variants.^[1]^

Although basaloid SCC is very rare, it is important to distinguish basaloid to other subtypes of SCCs, since the prognosis of basaloid subtype is considered poorer than that of other non-basaloid subtypes of SCCs.^[3,4]^ Basaloid SCCs are known to constitute a different histomolecular entity with non-basaloid SCCs, which contributes to intrinsic resistance to cytotoxic chemotherapy.^[5]^ For this reason, alternative targeted therapies may be needed.

Because of its rarity, there have been only few case reports describing the computed tomography (CT) findings of basaloid SCC.^[6–9]^ Therefore, the purpose of our study was to report the CT findings of basaloid SCC after the re-classification of SCC by the 2015 WHO classification of lung tumors.

## 2 Methods

### 2.1 Patient selection

The institutional review board (IRB) of Samsung Medical Center approved this retrospective study, and the requirement for patient consent to use clinical data was waived (IRB, file number 2021-05-062). We searched medical records from January 2016 to April 2021, and found 1347 patients who underwent curative lung resection surgery for SCC at Samsung Medical Center, a tertiary referral hospital located in Seoul, South Korea. By using the Medical Subject Heading terms “basaloid squamous cell carcinoma” by searching for these diagnoses from all electronic medical records, we found 12 (0.89%) patients (in records from for August 2016 to April 2021) who were surgically diagnosed with basaloid SCC of the lung among these 1347 patients.

### 2.2 Clinical and pathologic data

The clinicopathologic data of the patients were retrospectively reviewed for the following variables: age, sex, smoking history, and the presence of symptoms. In all 12 patients, the histopathologic reports were recorded. All tumors were staged on the basis of the malignant tumor staging system in the eighth edition of the American Joint Committee on Cancer TNM classification published in 2017.^[10]^ Pathologic specimens were examined and findings were reported using the latest edition of the WHO classification.^[1]^

### 2.3 Image acquisition

CT studies were performed using various helical CT scanners (mostly 16- to 64-MDCT scanners) from several vendors. The scanning parameters were 120 kVp and 170 to 200 mA under automatic exposure control; beam width, 10 to 20 mm; and rotation time, 0.3 to 0.4 seconds. Image data were reconstructed using standard soft-tissue algorithms; the data were reformatted with a section thickness of 2.5 to 3.0 mm for transverse images. Both enhanced and unenhanced CT scans were obtained. IV contrast medium injection was given in all patients: 1.5 mL/kg of body weight of iobitridol (300 mg I/mL; Xenetics, Guerbet) was injected at an infusion rate of 3 mL/s using a power injector (MCT Plus, Medrad). The interval time between the initiation of contrast injection and scanning was 45 seconds. The reconstructed images were then sent directly to a PACS (Centricity 3.0, GE Healthcare). Images were viewed on monitors in both mediastinal (width, 400 HU; level, 20 HU) and lung (width, 1500 HU; level, –700 HU) window settings.

### 2.4 Image interpretation

CT findings were reviewed by 2 radiologists with thoracic CT interpretation experience of 5 and 15 years, respectively, and decisions about CT findings were reached by consensus. The review included tumor size, attenuation value (in Hounsfield units) in unenhanced and contrast enhanced CT, net enhancement (attenuation difference between enhanced and unenhanced images), tumor location, presence of endobronchial growth, tumor margin, presence of cavitation, presence of internal profuse necrosis, presence of air-bronchogram, and presence of calcification. Tumor size was determined as the largest diameter on the transverse image where the equator of the tumor was scanned. When measuring tumor attenuation, the region of interest covering more than half of the tumor diameter was adopted on both enhanced and unenhanced images. The degree of enhancement of the lesion was also assessed subjectively and categorized as follows: mild, when the enhancement was similar to that of adjacent muscle; moderate, when the enhancement was higher than that of muscle, but lower than that of blood vessels; and marked, when the enhancement was approaching that of blood vessels. Tumor location was determined on CT images by dividing the lung approximately on the middle line into inner and outer regions. The inner region constitutes the central portion of the lung, which has a close relationship with the central airways. The outer region constitutes the peripheral portion of the lung, which has a close relationship with the pleura. For the peripherally located tumors, tumor margin was characterized as smooth, lobulated, or spiculated.

## 3 Results

### 3.1 Clinical findings

The clinical features of the 12 cases of basaloid SCC are summarized in Table 1. Seven patients were asymptomatic and were detected as incidental findings at routine health checkups of all patients. Five patients had symptoms such as cough (n = 3) and dyspnea (n = 2). The median age at the time of diagnosis was 71 years (range, 53–78 years). All patients were men. Five patients were current smokers, and the remaining 7 patients were ex-smokers (median, 37.5 pack-years). Majority of patients were at stage IA (58.3%). Three patients were stage IIB, and 1 each patient was stage IIA and IIIA.

**Table 1:** Clinical findings of 12 patients with basaloid squamous cell carcinoma.

Variable	Data
Age (yrs)	71 (53–78)^∗^
Sex	
Male	12 (92.3)
Female	0 (0.0)
Smoking history	
Current smoker	5 (41.7)
Ex-smoker	7 (58.3)
Never smoker	0 (0.0)
Smoking (pack-year)	37.5 (19–55)^∗^
Symptoms	
Yes	5 (41.7)
No	7 (58.3)
TNM stage	
IA	7 (58.3)
IB	0 (0.0)
IIA	1 (8.3)
IIB	3 (25.0)
IIIA	1 (8.3)
IIIB	0 (0.0)
IV	0 (0.0)

Unless otherwise indicated, data are number of patients with percentages in parentheses.

∗ Data are medians, with the range in parentheses.

### 3.2 Radiologic findings

**Figure 1:**
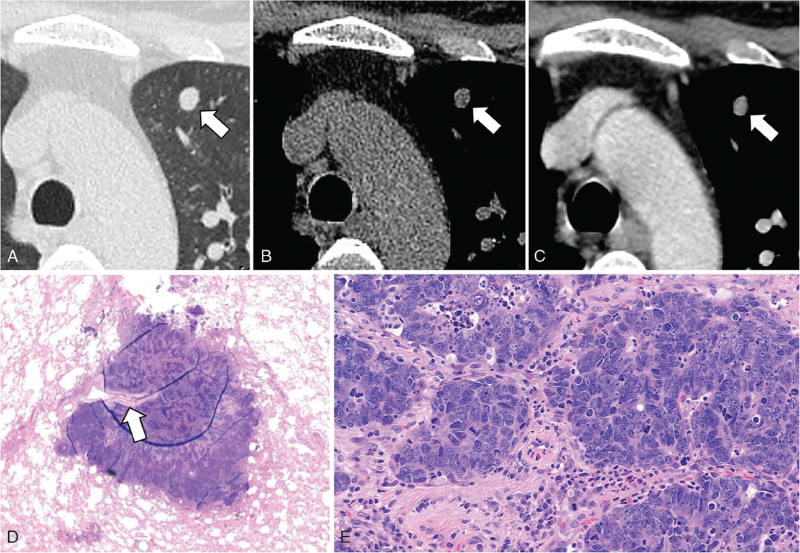
A 54-year-old man with basaloid squamous cell carcinoma of the lung. Lung window setting (A), non-enhanced (B), and enhanced (C) CT scans show well-enhancing nodule with smooth contour (arrow) in left upper lobe periphery. (D) Photomicrograph obtained at low magnification shows poorly differentiated well defined nodule without obstructing bronchiole (arrow), representing basaloid squamous cell carcinoma. (E) Photomicrograph obtained at medium-power magnification shows tightly packed nests of epithelial tumor cells with peripheral palisading which is typical pattern of basaloid squamous cell carcinoma. CT = computed tomography.

**Figure 2:**
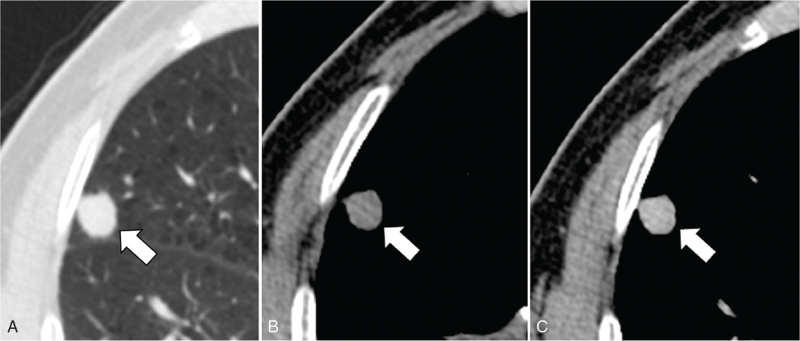
A 70-year-old man with basaloid squamous cell carcinoma of the lung. Unenhanced (A, B) and enhanced (C) CT scans show well-enhancing nodule with smooth contour (arrow) in right middle lobe periphery. CT = computed tomography.

**Figure 3:**
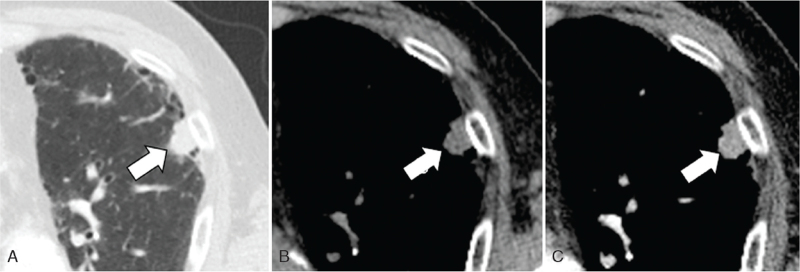
A 74-year-old man with basaloid squamous cell carcinoma of lung. (A) Lung window setting CT scan shows a nodule with lobulated contour (arrow) in left upper lobe subpleural area. Underlying pulmonary fibrosis of usual interstitial pneumonia pattern is noted. Non-enhanced (B) and enhanced (C) CT scans show moderate enhancement of the nodule (arrow) after contrast injection. CT = computed tomography.

**Figure 4:**
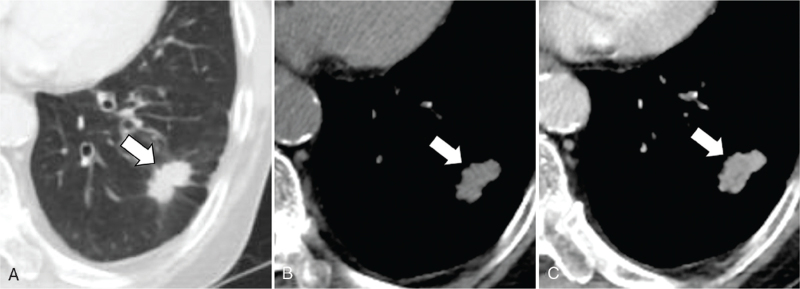
A 78-year-old man with basaloid squamous cell carcinoma of lung. Lung window setting (A), non-enhanced (B), and enhanced (C) CT scans show well-enhancing nodule with spiculated contour (arrow) in left lower lobe periphery. CT = computed tomography.

**Figure 5:**
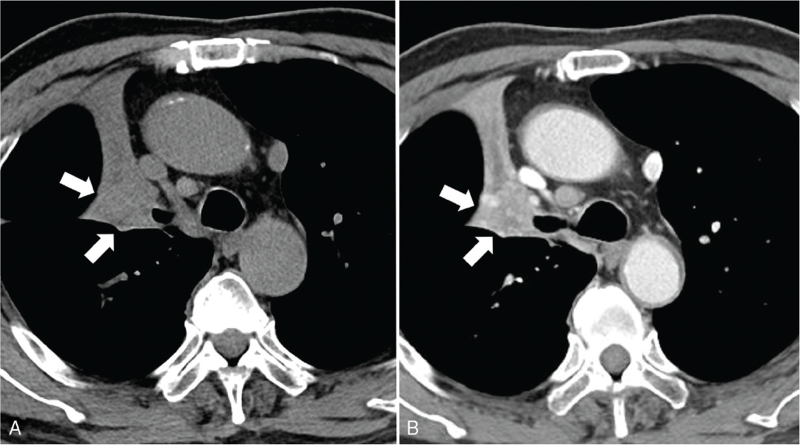
A 74-year-old man with basaloid squamous cell carcinoma of the lung. Unenhanced (A, B) and enhanced (B) CT scan shows enhancing mass obstructing right upper lobar bronchus with (arrows) with distal atelectasis. CT = computed tomography.

Image findings are summarized in Table 2. Radiologically, all tumors appeared as a solitary nodule (*n* *=* 9) or mass (*n* *=* 3). Tumor size ranged from 11 to 56 mm in the greatest dimension (median, 26 mm). Axial location was central in 5 patients, while 7 was in peripheral. Of the 7 patients whose tumors were located in the peripheral, margin of the tumor were smooth (*n* *=* 2) (Figs. 1 and 2), lobulated (*n* *=* 2) (Fig. 3), or spiculated (*n* *=* 3) (Fig. 4). Tumors showed low to intermediate attenuation on unenhanced CT scans with attenuation values ranging from 14.0 to 53.0 HU (median, 24.5 HU). After IV injection of contrast medium, enhancement was moderate, and the net enhancement value ranged from 15.8 to 71.8 HU (median, 36.4 HU) (Figs. 1–5). Endobronchial growth were seen in 5 patients and these patients accompanied obstructive pneumonia or atelectasis (Fig. 5). All of the 5 lesions showing endobroncial growth were centrally located. Internal profuse necrosis, cavitation, or calcifications were not seen. Of the patients whose tumors were located in the peripheral, air-bronchogram was seen in 1 patient.

**Table 2:** Image findings of basaloid squamous cell carcinoma.

Image findings	Data
Tumor size (mm)	26 (11–56)^∗^
Attenuation value in unenhanced CT (HU)	24.5 (14.0–53.0)^∗^
Attenuation value in contrast enhanced CT (HU)	60.5 (35.8–112.1)^∗^
Net enhancement value (HU)	36.4 (15.8–71.8)^∗^
Location	
Central	5 (41.7)
Peripheral	7 (58.3)
Endobronchial growth	
Yes	5 (41.7)
No	7 (58.3)
Margin	
Smooth	2 (28.6)
Lobulated	2 (28.6)
Spiculated	3 (42.9)
Cavitation	
Yes	0 (0.0)
No	12 (100.0)
Internal profuse necrosis	
Yes	0 (0.0)
No	12 (100.0)
Air-bronchogram	
Yes	1 (14.3)
No	6 (85.7)
Calcification	
Yes	0 (0.0)
No	12 (100.0)

Unless otherwise indicated, data are number of patients with percentages in parentheses. CT = computed tomography.

∗ Data are medians, with the range in parentheses.

### 3.3 Treatment and prognosis

Seven patients underwent lobectomy, 4 patients underwent wedge resection, and 1 patient had completion pneumonectomy due to previous operation. In all patients, mediastinal lymph node dissection was performed, and the resection was complete without positive resection margin. Mediastinal nodal metastases were found in 1 patient on surgical specimen evaluation: 1 lymph node metastasis was in the left interlobar node (11L).

Ten patients were alive without recurrence after a median follow-up period of 2 years (range, 18 days to 4.5 years). Three patients received adjuvant concurrent chemoradiation therapy (cisplantin and vinorelbine). None of these 3 patients had recurrent disease. One patient had lung to lung metastasis at 2 years after surgery. In another patient, tumor recurrence was found as mediastinal lymph node metastasis with hepatic metastasis at 10 months follow up. These 2 patients had peripherally located tumor with tumor size ranging from 1 to 2 cm, without lymph node metastasis at initial surgery.

## 4 Discussion

To the best of our knowledge, this study is the largest study to describe the CT findings of the basaloid SCCs. The results of the study showed that basaloid SCC of the lung manifested as solitary nodule or mass without cavitation or internal profuse necrosis within the tumor. Tumor showed moderate enhancement and was located either peripheral or central compartment of the lung. Peripherally located tumor was shown as smooth, lobulated, or spiculated. Centrally located tumor showed endobronchial growth resulting obstruction of bronchus. We found 3 reports of 4 patients regarding image findings in the English-literature of basaloid SCCs of the lung.^[6–9]^ Most of these reports, like our study, showed solitary tumor with mild to moderate enhancement with either central or peripheral location on CT. Kim et al,^[7]^ have reported 2 cases that showed prominent internal necrosis of the tumor and 1 case showed thin walled cavitation of the tumor. However, none of the case in our study showed cavitation of the tumor.

A unique CT finding noted in our study group is the absence of cavitation. Cavitation is seen approximately 10% to 22% of lung cancer and especially common in SCC.^[11–14]^ Cavitation occurs up to 17% to 40% in SCC^[14,15]^ and 80% to 82% of cavitating lung tumors are reported to be SCCs.^[11,16]^ Cavitation is formed by drainage of substances after tumor necrosis. There are several mechanisms that explain tumor necrosis in primary lung cancer. Among them the representative theory is that in the usual course of tumor necrosis, the vessel wall is invaded by tumor and tumor accesses to perivascular spaces and it spreads interstitially or through the alveolar spaces via the pores of Cohn. Eventually the pulmonary architecture is destroyed and large central avascular necrosis ensues.^[11,14,17]^ In histology, basaloid SCC may show centrilobular foci of necrosis,^[2,18]^ but we believe that prominent necrosis which is radiologically evident is not a common finding due to its histopathologic nature. In our theory, those peripheral palisading with radially arranged cells at the periphery of lobules in basaloid SCC would act as a physical barrier to block the propagation of tumor invasion. Another important mechanism of tumor necrosis is “stenotic abscess” due to infection and breakdown of the lung parenchyma distal to bronchial obstruction caused by the tumor growth.^[11]^ Histologically, basaloid SCC grows invasively in a finger-like fashion from the bronchial lining. As this growth pattern of cells gradually narrows rather than completely blocks the bronchi, we think that it may cause less necrosis compared to other bronchial origin tumors. In correlation with our study, previous literature reporting basaloid SCCs, most of tumors have exhibited nodular or mass lesions without cavity formation.^[3,6–9]^ Yamada et al^[19]^ have reported an unusual case of basaloid SCC with cavity that showed coagulative necrotic foci of pre-existing alveolar wall in the cancer cavity junction. They assumed that this might have occurred in the same way as that cavities were created in rare cases in adenocarcinoma. The theory is as follows: adenocarcinoma cells initially develop in alveolar wall and grow toward bronchiole, and next formed a unidirectional check-valve owing to lack of cartilage in bronchiole; the accumulations of gases enter alveoli; the alveoli rupture and fuse into cavity with separation; and finally, the cavity gradually gets larger and larger with the increased inner pressure.^[20]^ We think that the same principle can explain a case of thin walled cavity shown in case report of Kim et al^[7]^ that was mentioned above.

Meanwhile in molecular basis, Onn et al^[14]^ reported that patients with non-small cell lung cancer with cavitary lesions are likely to have tumor epidermal growth factor receptor overexpression. However, epidermal growth factor receptor expression in basaloid SCC has not been studied so far.

In our study, percentage of tumors located in the peripheral compartment of the lung was relatively higher than those in central portion (58.3% and 41.7% each). It is well known fact that localization of SCC is predominantly central and about one third of SCC are peripherally located.^[21]^ However, incidence of peripheral SCC seems to be increasing and referring to recent reports, peripheral SCC is reported up to 40% to 55% of all lung SCC^[22–24]^ in correlation with our study.

Owing to the small cohort, patient prognosis cannot be exactly compared with other SCC. In our case series, the treatment strategy for basaloid SCC was not different from that of primary non-small cell lung cancers. In our study, 1 of the 12 (8.3%) patient had mediastinal lymph node metastasis, similar to previous report.^[25]^ Two patients (16.7%) had recurrent disease, but in the remaining 10 patients were alive without recurrence after a median follow-up period of 2.0 years (range, 18 days to 4.5 years). Although there is some controversy,^[4,26]^ prognosis of basaloid SCC remains poor.^[2,3,5]^ A 5-year survival rate of only 15% has been reported even in stage I and II of basaloid SCC.^[4]^ Since large portion of our study cohort were asymptomatic patients whose cancers were found in routine health checkup (58.3%, 7 of 12 patients), there is possibility that the survival rate is better estimated than the previously known survival rate.

Several drawbacks limited this study. First, our study has a retrospective design and a small number of patients because we only included data from 2016, since basaloid SCCs was not clearly established as SCC or large cell carcinoma before 2015. Further studies with larger sample sizes and comparison of image finding with non-basaloid SCC are required to validate our results. Second, our follow up period was too short by median of only 2 years. Larger scale with long term follow up study is needed to better understand the tumor behavior and prognosis.

## 5 Conclusions

In conclusion, basaloid SCC of the lung manifested as solitary nodule or mass with moderate enhancement. Tumor was located either peripheral or central compartment of the lung and cavitation was absent in CT.

## Author contributions

All authors read and final approval of the manuscript.

**Conceptualization:** Hyun Jung Yoon, Tae Jung Kim, Yoon Ki Cha, C.H. Kim.

**Data curation:** Chu Hyun Kim, Yoon Ki Cha, J.H. Kim.

**Formal analysis:** Chu Hyun Kim, Yoon Ki Cha.

**Methodology:** Yoon Ki Cha, Hyun Jung Yoon.

**Project administration:** Jung Hee Lee.

**Resources:** Jun Ho Kim, Joungho Han, Yoon Ki Cha.

**Supervision:** Joungho Han, Jun Ho Kim, Myung Jin Chung.

**Visualization:** Tae Jung Kim, Myung Jin Chung, Yoon Ki Cha.

**Writing – original draft:** Yoon Ki Cha, Chu Hyun Kim.

**Writing – review& editing:** Chu Hyun Kim, Yoon Ki Cha, Joungho Han, Myung Jin Chung, Jung Hee Lee, Tae Jung Kim, Hyun Jung Yoon
